# Is slowness a better discriminator of disability than frailty in older adults?

**DOI:** 10.1002/jcsm.12810

**Published:** 2021-09-29

**Authors:** Dayane Capra de Oliveira, Roberta de Oliveira Máximo, Paula Camila Ramírez, Aline Fernanda de Souza, Mariane Marques Luiz, Maicon Luis Bicigo Delinocente, Marcos Hortes Nisihara Chagas, Andrew Steptoe, Cesar de Oliveira, Tiago da Silva Alexandre

**Affiliations:** ^1^ Postgraduate Program in Physical Therapy Federal University of Sao Carlos São Carlos Brazil; ^2^ Escola de Fisioterapia Universidad Industrial de Santander Bucaramanga Colômbia; ^3^ Postgraduate Program in Gerontology Federal University of Sao Carlos São Carlos Brazil; ^4^ Gerontology Department Federal University of Sao Carlos São Carlos Brazil; ^5^ Department of Epidemiology and Public Health University College of London (UCL) London England

**Keywords:** Frailty, Disability, Trajectories, Older adults, Slowness

## Abstract

**Background:**

The trajectory of incident disability that occurs simultaneously with changes in frailty status, as well as how much each frailty component contributes to this process in the different sexes, are unknown. The objective of this study is to analyse the trajectory of the incidence of disability on basic and instrumental activities of daily living (BADL and IADL) as a function of the frailty changes and their components by sex over time.

**Methods:**

Longitudinal analyses of 1522 and 1548 of the English Longitudinal Study of Ageing study participants without BADL and IADL disability, respectively, and without frailty at baseline. BADL and IADL were assessed using the Katz and Lawton Scales and frailty by phenotype at 4, 8, and 12 years of follow‐up. Generalized mixed linear models were calculated for the incidence of BADL and IADL disability, as an outcome, using changes in the state of frailty and its components, as the exposure, by sex in models fully adjusted for sociodemographic, behavioural, biochemical, and clinical characteristics.

**Results:**

The mean age, at baseline, of the 1522 eligible individuals free of BADL and free of frailty was 68.1 ± 6.2 years (52.1% women) and of the 1548 individuals free IADL and free frailty was 68.1 ± 6.1 years (50.6% women). Women who became pre‐frail had a higher risk of incidence of disability for BADL and IADL when compared with those who remained non‐frail (*P* < 0.05). Men and women who became frail had a higher risk of incidence of disability regarding BADL and IADL when compared with those who remained non‐frail (*P* < 0.05). Slowness was the only component capable of discriminating the incidence of disability regarding BADL and IADL when compared with those who remained without slowness (*P* < 0.05). Weakness and low physical activity level in men and exhaustion in women also discriminated the incidence of disability (*P* < 0.05).

**Conclusions:**

Slowness is the main warning sign of functional decline in older adults. As its evaluation is easy, fast, and accessible, screening for this frailty component should be prioritized in different clinical contexts so that rehabilitation strategies can be developed to avoid the onset of disability.

## Introduction

Frailty is a clinical syndrome characterized by reductions in the homeostatic reserve and resistance to stressors, resulting from the cumulative decline of multiple physiological systems, which increases the risk of negative outcomes in older adults.^1^ This condition is not synonymous with disability and can be reversed or attenuated by interventions if detected early.^2^ However, this is a bidirectional relationship, as studies have demonstrated that the frailty process increases the risk of the incidence of disability and disability increases the risk of the incidence of frailty.^3^, ^4^, ^5^, ^6^

Cross‐sectional studies have shown that disability is associated with frailty.^7^ In a study with four and a half years of follow‐up, Pollack *et al*..^4^ investigated 5086 older men and found that those with limitations regarding activities of daily living were at greater risk of developing frailty. Liu *et al*.^5^ confirmed this association in a meta‐analysis. In contrast, Aguilar‐Navarro *et al*.^6^ conducted an 11 year follow‐up study involving 5644 older adults and found that frailty was a risk factor for the incidence of disability. Similarly, Makizako *et al*.^3^ showed that pre‐frailty increased both the risk of disability and worse disability trajectories in the final year of life in frail older adults.^8^ However, there are no studies analysing the trajectory of the incidence of disability regarding basic and instrumental activities of daily living (BADL and IADL, respectively) as a function of the trajectory of the incidence of frailty and its components by sex.

Differences between sexes regarding the frailty process and the development of disability have been widely investigated, with studies showing consistent results. For example, men resist frailty for a longer time, but when the incidence of the syndrome occurs, they survive for a shorter period of time.^9^ In contrast, women become frail more quickly and live for a longer time with the syndrome.^10^ The prevalence and incidence of associated diseases is also higher among women, leading to a greater risk of disability, whereas men tend to have diseases that lead more quickly to death.^11^, ^12^ Therefore, the mechanisms of frailty and disability onset may be influenced by different factors in older men and women.^9^, ^13^

Therefore, the aim of the present study was to test the following hypotheses: (i) the trajectories of the incidence of BADL and IADL disability are worse in individuals who become frail compared with those who become pre‐frail or remain non‐frail; (ii) the occurrence of frailty components related to musculoskeletal function are good discriminators of a greater incidence of BADL and IADL disability; and (iii) there are differences between the sexes in these associations.

## Methods

### Study population

The data used in this investigation were from the English Longitudinal Study of Ageing (ELSA), which is a longitudinal panel study of older adults living in England aged 50 years or older that began in 2002.^14^ A detailed description of the study can be found elsewhere.^14^

The ELSA sample in 2004, when anthropometric data and physical performance were collected for the first time, was composed of 6183 individuals aged 60 years or older. Among these individuals, 2138 and 2180 were free of BADL and IADL disability and frailty, respectively, at baseline. A total of 616 and 632 individuals were excluded from the sample for the BADL and IADL trajectory analysis, respectively, due to a lack of information on covariates, resulting in a final analytical sample of 1522 and 1548 individuals. The participants were re‐evaluated after 4, 8 and 12 years of follow‐up.

All participants provided informed consent, and ethical approval for ELSA was obtained from the Multicentre Research and Ethics Committee (MREC/01/2/91).

### Basic activities of daily living

Basic activities of daily living were evaluated using the modified Katz index^15^ (bathing, feeding, walking, transferring, dressing, and toileting). Although continence is part of these items, it does not necessarily imply a physical limitation and was, therefore, not included in the present analysis. Only individuals who did not have difficulties performing any BADL at baseline were included in the present study. The incidence of difficulties in BADL in the 12 year follow‐up period was analysed and scores ranged from 0 to 6.

### Instrumental activities of daily living

Instrumental activities of daily living were evaluated using the adapted Lawton scale^16^ (housekeeping, doing laundry, preparing meals, using transportation, shopping, using the telephone, handling finances, and managing medications). Only individuals who did not have difficulties performing any IADL at baseline were included in the present study. The incidence of difficulties on IADL in the 12 year follow‐up period was analysed and scores ranged from 0 to 7.

### Frailty

Frailty was analysed using the adapted Fried *et al*.^1^ model. Unintentional weight loss was defined as the loss of 5% of body weight in the interval between each interview or by a body mass index (BMI) < 18.5 kg/m^2^ ^17^ at baseline. Exhaustion was defined as a positive response to either of the two statements ‘Felt that everything I did was an effort in the last week’ or ‘Could not get going in the last week’ from the Center for Epidemiologic Studies Depression Scale (CES‐D).^18^ Weakness: lowest quintile of grip strength stratified by sex in each quartile of BMI. Slowness: lowest quintile of walking speed based on the average of two measurements (2.4 m) stratified by height (mean) and sex. The low physical activity level (LPAL) was determined based on the frequency and intensity with which the participants practiced vigorous, moderate, and mild physical activity (more than one per month, once per week, one to three times per week, or never). Those who reported never performing moderate intensity physical activity were considered positive for the LPAL.^19^ Participants with three or more the components above described were considered frail. Those with one or two components as pre‐frail and those with none non‐frail. Only non‐frail individuals at baseline were included in the present investigation.

### Covariates

Factors reported in the literature as associated with the incidence of frailty and BADL/IADL disabilities were included as covariates.^11^, ^12^, ^13^ The sociodemographic characteristics were age (years), marital status (with/without conjugal life), skin colour (white/non‐white), household total wealth (quintiles) and level of education (0–11, 12–13, and >13 years).

The behavioural characteristics were frequency of alcohol intake (never, once per week, two to six times per week, daily, or not declared),^20^ smoking (non‐smoker, ex‐smoker, or smoker)^20^ and level of physical activity.^21^ Participants reported their frequency (once per week, more than once per week, one to three times per month, and never) of mild, moderate, or vigorous intensity physical activity. They were then categorized into three groups according to their responses related to the intensity and frequency of physical activity in: (0) vigorous/moderate; (1) low (at least once a week) or (2) sedentary lifestyle (no weekly activity).

Health conditions were obtained through doctor diagnosed self‐reports of stroke, heart disease, cancer, lung disease, joint disease, osteoporosis, falls in the previous 12 months, and dementia. Systemic arterial hypertension and diabetes were identified by self‐reports of a medical diagnosis and/or systolic blood pressure ≥ 130 mmHg and/or diastolic blood pressure ≥ 85 mmHg^22^ and glycated haemoglobin ≥ 6.5%, respectively.^23^ Vision and hearing were classified as good, fair, or poor. Depressive symptoms were defined by CES‐D score ≥ 4.^18^ Based on BMI, the individuals were classified as normal (≥18.5 and <25 kg/m^2^), undernourished (<18.5 kg/m^2^), overweight (≥25 and <30 kg/m^2^) or obese (≥30 kg/m^2^).^17^ Memory was based on the summation of immediate and delayed‐recall results from a 10 word‐list learning test (score range: 0–20), with higher scores denoting better cognitive function.^24^

Blood samples were collected for biochemical analysis during the nurses visits after the participants had remained for 5 h without ingesting foods or beverages, besides water. Further information on the laboratory analyses are found elsewhere.^25^ The following were determined: triglycerides (≥150 mg/dL), total cholesterol (≥200 mg/dL), HDL (<40 mg/dL for men and <50 mg/dL for women), LDL (≥100 mg/dL),^26^ fibrinogen (>3.8 g/L),^27^ and anaemia (<12 mg/dL for women and <13 mg/dL for men).^28^

### Statistical analyses

Baseline characteristics were expressed as means, standard deviation (SD) and proportions. Differences among individuals free of disability and frailty, stratified by sex, at baseline and differences between included and excluded individuals (due to missing information), stratified by sex, were evaluated using the *χ* ^2^ test and Student's *t*‐test. A *P* value < 0.05 was considered indicative of statistical significance.

Generalized linear mixed models were created using the XTMIXED command in Stata15® SE (Stata Corp, College Station, TX, USA) to estimate the trajectories of the incidence of BADL and IADL disability separately and stratified by sex. This analytical approach was chosen as the best modelling technique for unbalanced data and repeated measures, enabling the analysis of changes in a time‐dependent variable as well as enabling time‐dependent changes in the association between variables.^29^, ^30^

There were no differences in the intercept on the incidence trajectories of BADL and IADL disability, because we excluded individuals with disability and frailty at baseline. Therefore, the model presents the slope, which indicates the incidence trajectories of disability regarding BADL and IADL per year, stratified by sex, according to the three frailty groups (non‐frail individuals who remained non‐frail, non‐frail individuals who became pre‐frail and non‐frail individuals who became frail) as well as by each frailty component occurrence over time.

Variables with a *P* value ≤ 0.20 in the univariate analyses were selected for the multiple models using the stepwise forward method.^31^ The results for the disability trajectories, stratified by sex, were compared using *β* coefficients and respective 95% confidence intervals, considering the group that remained non‐frail as the reference. For the models in which the components were analysed separately, the weakness, LPAL, slowness, exhaustion, and unintentional weight loss were classified as dichotomous variables (with those who remained without the component considered the reference group).

## Results

Among the 1522 participants free of BADL disability and frailty at baseline, 72.2%, 54.0%, and 35.2% were re‐evaluated at 4, 8, and 12 years of follow‐up. Among the 1548 participants free of IADL disability and frailty at baseline, 72.7%, 53.9%, and 35.3% were re‐evaluated at 4, 8, and 12 years of follow‐up.

The mean age of men and women free of BADL or IADL disability and free of frailty at baseline was 68 years. More men than women were married, ingested alcohol on a daily basis, were ex‐smokers and were overweight. The majority of women did not have a conjugal life, had lower schooling, a higher frequency of hypercholesterolemia and higher levels of LDL cholesterol, greater frequencies of joint disease, osteoporosis and falls, a greater proportion of hearing perceived as good, and a higher mean memory score compared with the men (*Tables* *1* and *2*). Participants who were excluded, due to missing information at baseline, reported a greater frequency of ‘not declared’ alcohol intake, smoked more, or never smoked and had a greater frequency of hypertriglyceridemia compared with the included individuals (Supporting Information, *Tables* *S1* and *S2*).

**Table 1:** Socio‐economic, behavioural, and biochemical characteristics of individuals without BADL and IADL disability and frailty of ELSA (2004–05)

	BADL			IADL		
	Men	Women	Total	Men	Women	Total
	(*n* = 729) 47.9%	(*n* = 793) 52.1%	(*n* = 1522)	(*n* = 764) 49.4%	(*n* = 784) 50.6%	(*n* = 1548)
Socio‐economic variables						
Age, years (SD)	68.2 ± 6.1	68.1 ± 6.2	68.1 ± 6.2	68.2 ± 6.0	68.0 ± 6.2	68.1 ± 6.1
Without conjugal life (yes), %	17.7*	35.7*	27.1	17.7*	35.5*	26.7
Non‐white skin colour (yes), %	0.8	1.4	1.1	0.8	1.1	1.0
Family wealth (quintiles), %						
Highest quintile	30.3	29.1	29.7	30.4	29.6	30.0
2nd quintile	26.2	24.8	25.5	25.5	24.7	25.1
3rd quintile	22.5	19.6	20.9	23.2	19.8	21.4
4th quintile	13.5	15.6	14.6	13.5	15.3	14.4
Lowest quintile	6.7	9.5	8.2	6.7	9.1	7.9
Not declared	0.8	1.4	1.1	0.7	1.5	1.2
Schooling, %						
>13 years	37.6*	24.1*	30.6	37.4*	24.2*	30.8
12–13 years	24.8	23.7	24.2	24.5	24.0	24.2
0–11 years	37.6*	52.2*	45.2	38.1*	51.8*	45.0
Behavioural variables						
Alcohol intake, %						
≤1 day per week	7.6*	18.5*	13.2	8.2*	18.5*	13.5
2–6 days per week	40.7	46.9	44.0	41.0	47.2	44.1
Daily	45.4*	30.4*	37.6	45.0*	30.4*	37.6
Not declared	6.3	4.2	5.2	5.8	3.9	4.8
Smoking, %						
Non‐smoker	30.9*	52.3*	42.1	31.1*	52.3*	41.9
Ex‐smoker	60.8*	40.7*	50.3	60.5*	40.8*	50.5
Smoke	8.3	7.0	7.6	8.4	6.9	7.6
Active lifestyle, %^a^						
Low	100.0	100.0	100.0	100.0	100.0	100.0
Biochemical characteristics						
Triglycerides (≥150 mg/dL), %	40.5	35.2	37.7	41.1	35.3	38.2
Total cholesterol (≥200 mg/dL), %	65.2*	83.7*	74.8	64.9*	83.5*	74.3
HDL (<40 mg/dL M; <50 mg/dL W), %	11.9	11.2	11.6	12.6	11.9	12.2
LDL (≥100 mg/dL), %	81.8*	90.0*	86.1	80.9*	90.6*	85.8
Fibrinogen (>3.7 g/L), %	19.1	22.1	20.6	19.0	22.7	20.9
Anaemia (<13 g/dL M; <12 g/dL W), %	3.4	2.6	3.0	3.7	2.5	3.1

BADL, basic activities of daily living; ELSA, English Longitudinal Study of Ageing; HDL, high‐density lipoprotein; IADL, instrumental activities of daily living; LDL, low‐density lipoprotein; M, men; W, women.

Data expressed as mean, standard deviation and proportion

^a^ All individuals with a sedentary lifestyle were excluded at baseline, and there were no individuals in the group of vigorous/moderate physical activity.

* Significant difference between sexes (*P* < 0.05, *χ* ^2^test).

**Table 2:** Clinical characteristics of individuals without BADL and IADL disability frailty of ELSA (2004–05)

	BADL			IADL		
	Men (*n* = 729) 47.9%	Women (*n* = 793) 52.1%	Total (*n* = 1.522)	Men (*n* = 764) 49.4%	Women (*n* = 784) 50.6%	Total (*n* = 1.548)
Clinical conditions						
Stroke (yes), %	2.9	1.6	2.2	3.0	1.1	2.1
Heart disease (yes), %	21.8	16.9	19.2	22.0	17.0	19.4
Cancer (yes), %	6.7	8.3	7.6	6.4	8.3	7.4
Lung disease (yes), %	13.3	14.2	13.8	13.5	14.0	13.8
Joint disease (yes), %	23.5*	32.3*	28.1	24.2*	33.3*	28.8
Osteoporosis (yes), %	1.5*	8.9*	5.4	1.6*	8.7*	5.2
Falls (yes), %	16.2*	30.0*	23.4	17.0*	29.8*	23.4
Dementia (yes), %	0.4	0.1	0.3	0.1	0.1	0.1
Hypertension (yes), %	73.5	72.4	72.9	74.5	72.1	73.3
Diabetes (yes), %	8.8	6.2	7.4	9.7	6.0	7.8
Perception of hearing, %						
Good	76.2*	87.8*	82.2	76.3*	88.3*	82.4
Fair	19.6*	10.6*	14.9	19.9*	9.9*	14.8
Poor	4.2*	1.6*	2.9	3.8	1.8	2.8
Perception of vision, %						
Good	94.4	93.3	93.8	93.7	93.2	93.5
Fair	4.5	5.6	5.1	5.5	5.6	5.5
Poor	1.1	1.1	1.1	0.8	1.2	1.0
Depressive symptoms, %						
No	98.5	97.5	98.0	98.8	97.3	98.1
Yes	1.1	2.3	1.7	0.8	2.4	1.6
Not declared	0.4	0.2	0.3	0.4	0.3	0.3
Mean recall score, points (SD)	9.9 ± 2.9*	10.7 ± 3.2*	10.3 ± 3.1	9.9 ± 2.9*	10.8 ± 3.1*	10.3 ± 3.0
BMI (kg/m^2^), %						
Normal weight (≥18.5 and <25)	25.2*	34.7*	30.2	24.2*	33.3*	28.8
Overweight (≥25 and <30)	54.3*	41.6*	47.7	53.2*	42.2*	47.6
Obesity (≥30)	20.5	23.7	22.1	22.6	24.5	23.6

BADL, basic activities of daily living; BMI, body mass index; CES‐D, Center for Epidemiological Studies Depression Scale; ELSA, English Longitudinal Study of Ageing; IADL, instrumental activities of daily living.

Data expressed as mean, standard deviation, and proportion.

* Significant difference between sexes (*P* < 0.05, *χ* ^2^test).

The estimated parameters for the incidence of BADL and IADL disability, separately, over time (slope), as a function of changes in frailty status and the occurrence of each component in 12 years of follow‐up are shown in *Table* *3*, respectively.

**Table 3:** GLMMs estimates for incidence of BADL and IADL disability by sex as a function of the frailty changes and their components in 12 year follow‐up

	BADL		IADL	
	Men (*n* = 729)	Women (*n* = 793)	Men *n* = 764)	Women (*n* = 784)
Frailty	Estimated parameters (95% CI)			
Slope				
Time, years	0.008 (−0.060 to 0.075)	−0.104 (−0.166 to −0.043)*	−0.042 (−0.122 to 0.039)	−0.128 (−0.189 to −0.067)**
Time × NF/NF	Reference	Reference	Reference	Reference
Time × NF/PF	0.005 (−0.001 to 0.011)	0.009 (0.004 to 0.015) *	−0.003 (−0.008 to 0.002)	0.006 (0.000 to 0.012)*
Time × NF/F	0.047 (0.034 to 0.061)**	0.016 (0.003 to 0.028)*	0.031 (0.019 to 0.044)**	0.045 (0.032 to 0.058)**
Components	Estimated parameters (95% CI)			
Slope				
Time, years	−0.049 (−0.145 to 0.048)	−0.085 (−0.147 to −0.023)*	−0.085 (−0.163 to −0.007)*	−0.130 (−0.183 to −0.078)**
Time × LPAL (yes)	0.016 (0.005 to 0.026)*	−0.004 (−0.013 to 0.005)	0.016 (0.007 to 0.025)**	0.028 (0.020 to 0.036)**
Time × Slowness (yes)	0.021 (0.012 to 0.029)**	0.024 (0.016 to 0.033)**	0.010 (0.003 to 0.018)*	0.012 (0.004 to 0.020)*
Time × Weakness (yes)	0.009 (0.001 to 0.016)*	—	0.008 (0.002 to 0.015)*	—
Time × Exhaustion (yes)	0.007 (−0.001 to 0.015)	0.019 (0.013 to 0.026)**	0.007 (−0.001 to 0.014)	0.023 (0.017 to 0.030)**

BADL, basic activities of daily living; CI, confidence interval; F, frail; GLMMs, generalized linear mixed models; IADL, instrumental activities of daily living; LPAL, low physical activity level; NF, non‐frail; PF, pre‐frail.

ELSA (2004/2005–2016/2017). In the trajectories of the incidence of BADL and IADL disability analysed, there was no difference in the intercept, as we excluded individuals with disability and frailty at baseline. (—) values did not enter the final model. BADL and frailty criteria model for men adjusted by perception of vision and hearing, falls, schooling, BMI (kg/m^2^), and lung disease. BADL and frailty criteria model for women adjusted by age, stroke, low‐density lipoprotein (LDL), triglycerides, anaemia, perception of hearing, joint disease and BMI (kg/m^2^). IADL and frailty criteria model for men adjusted by stroke, perception of hearing, falls, BMI (kg/m^2^), triglycerides, alcohol consumption, schooling, and osteoporosis. IADL and frailty criteria model for women adjusted by age, perception of vision, stroke, triglycerides, anaemia, and joint disease. BADL and components (LPAL, slowness, and weakness) model for men adjusted by falls, schooling, lung disease, LDL, stroke, and marital status. BADL and components (slowness and exhaustion) model for women adjusted by age, stroke, LDL, anaemia, perception of hearing, BMI (kg/m^2^) and diabetes. IADL and components (LPAL, slowness, and weakness) model for men adjusted by age, stroke, perception of hearing, falls, BMI (kg/m^2^), triglycerides, and schooling. IADL and components (LPAL, slowness and exhaustion) model for women adjusted by age, dementia, perception of vision, stroke, BMI (kg/m^2^), joint disease, osteoporosis, and falls.

* *P* < 0.05.

** *P* < 0.01.

**Figure 1:**
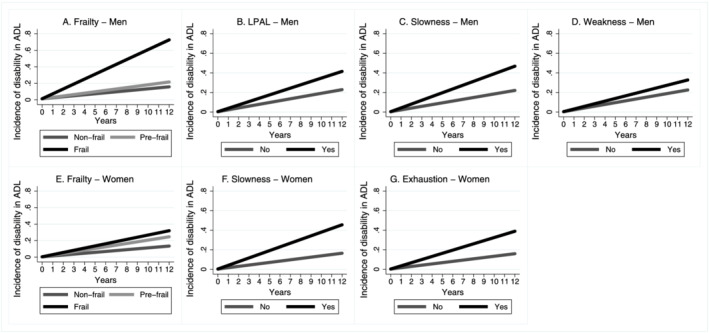
Incidence trajectory of disability in BADL by sex according to frailty status in 12‐year follow‐up, ELSA, England, 2004/2005–2016/2017. (*A*) BADL predictions and frailty criteria—men (*n* = 729) adjusted by perception of vision and hearing, falls, schooling, BMI (kg/m^2^), and lung disease. (*B*, *C*, and *D*) BADL predictions components—men (*n* = 729) adjusted for falls, schooling, lung disease, low‐density lipoprotein (LDL), stroke (stroke), and marital status. (*E*) BADL predictions and frailty criteria—women (*n* = 793) adjusted for age, stroke, LDL, triglycerides, anaemia, perception of hearing, joint disease and BMI (kg/m^2^). (*F* and *G*) BADL predictions components—women (*n* = 793) adjusted for age, stroke, LDL, anaemia, perception of hearing, BMI (kg/m^2^), and diabetes. ADL, activities of daily living; LPAL, low physical activity level.

**Figure 2:**
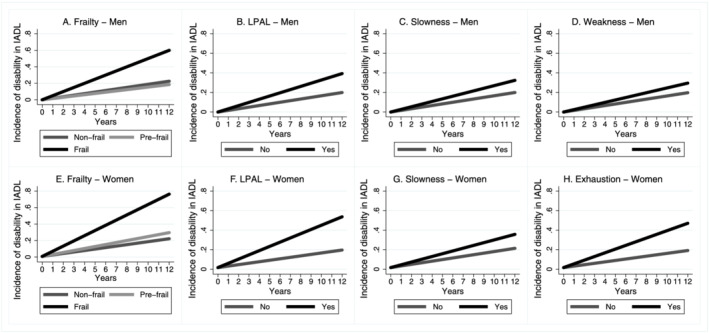
Incidence trajectory of disability in IADL by sex according to frailty status in 12 year follow‐up, ELSA, England, 2004/2005–2016/2017. (*A*) IADL predictions and frailty criteria—men (*n* = 764) adjusted by stroke, perception of vision, falls, BMI (kg/m^2^), triglycerides, alcohol consumption, education, and osteoporosis. (*B*, *C*, and *D*) IADL predictions components—men (*n* = 764) adjusted for age, stroke, perception of vision, falls, BMI (kg/m^2^), triglycerides, and schooling. (*E*) IADL predictions and frailty criteria—women (*n* = 784) adjusted for age, perception of vision, stroke, triglycerides, anaemia, and joint disease. (*F*, *G*, and *H*) IADL predictions components—women (*n* = 784) adjusted for age, dementia, perception of vision, stroke, BMI (kg/m^2^), joint disease, and osteoporosis. IADL, instrumental activities of daily living; LPAL, low physical activity level.

Our findings showed that to become pre‐frail during the follow‐up period was related to an increase in the risk of the incidence of BADL and IADL disability in women but not in men. Men and women who became frail were at greater risk of the incidence of BADL and IADL disability compared with those who remained non‐frail (reference) (*Table* *3* and *Figures* *1* and *2*).

The occurrence of LPAL, slowness, and weakness increased the risk of BADL and IADL disability in men (*Table* *3* and *Figures* *1* and *2*). In women, the occurrence of slowness and exhaustion increased the risk of the incidence of BADL disability. LPAL, slowness, and exhaustion increased the risk of the incidence of IADL disability in women (*Table* *3* and *Figures* *1* and *2*).

## Discussion

Our main findings showed, for the first time, that women who became pre‐frail and men and women who became frail had worse trajectories of incidence of disability then those who remained non‐frail. However, slowness was the only frailty component capable of discriminating the incidence of BADL and IADL disability in men and women. Besides slowness, weakness, and LPAL were good discriminators of incidence of disability in men. Exhaustion discriminated better the incidence of disability in women.

The mechanisms of becoming frail and disabled share similar physiopathological mechanisms in older adults but differ between men and women.^9^, ^11^, ^12^ In the present study, the incidence of pre‐frailty was a determinant of the incidence of disability only in women. Recent studies have shown that women have a greater physiological reserve than men and, consequently, withstand a greater number of changes in multiple systems, especially the musculoskeletal (osteoarticular), immunological and neuroendocrine systems.^9^ However, the accumulation of these deficits exerts a greater influence on the emergence of frailty and disability in women, as confirmed in the present investigation.

The present study also showed that frailty was a risk factor to the incidence of BADL and IADL disability in both sexes. On the other hand, it seems that men withstand physiological dysregulations more.^32^ This does not mean that men have a more competent physiological system than women, but rather that men have a greater capacity to maintain homeostatic equilibrium below a clinical threshold.^10^ However, when men develop the phenotype and disabilities, these outcomes can be more advanced and rapidly progress to mortality.^10^, ^11^, ^12^, ^13^, ^32^, ^33^

Independently of sex, the process of becoming frail and disabled is dynamic, complex, and mediated by biological, behavioural, and social factors.^11^, ^12^, ^13^ Therefore, an individual's level of exposure to these factors entails particularities that can either increase or diminish the risk of frailty and disability.^33^ In the present study, we identified slowness as the only frailty component capable of discriminating the incidence of disability regarding BADL and IADL in both sexes. This finding may contribute to a better understanding of the frailty–disability process.

Changes in domains considered central to the maintenance of mobility capacity, such as the central nervous, osteoarticular, sensory‐perception, and musculoskeletal systems, have been associated with a greater risk of impaired mobility, evidenced by slow gait speed.^34^ Slowness is considered an important warning sign of both functional decline and an increased risk of death in older adults, contributing to the emergence of the phenotype and the development of functional disability.^3^, ^34^, ^35^, ^36^ Following up 14 081 older people (65 years of age or older) for 29.5 months, Shimada *et al*.^35^ found that, independently of frailty status (pre‐frail or frail) at baseline, those with a slower gait were at greater risk for the incidence of disability compared with non‐frail individuals. Thus, among the components of frailty, slowness seems to be the best discriminator of the incidence of disability regarding BADL and IADL in both sexes.

Low physical activity level was also associated to the incidence of disability in both sexes in the present investigation. This finding is in line with the results of cross‐sectional study involving community‐dwelling older people, which found significant associations between disability and both slowness and LPAL.^36^ The effects of LPAL on functioning may be influenced by the reduction in muscle mass and strength, resulting in a poorer physical performance and exerting an effect on gait speed.^3^, ^37^

Other components may also increase the risk of the incidence of disability in men and women. However, the identification of slowness as the best discriminator of the risk of the incidence of functional disability reflects changes in multiple systems interrelated with other components of frailty also capable of exerting an influence on the installation of disability. In the present study, the incidence of weakness was associated with the incidence of disability in men. The reduction in neuromuscular strength, which is the result of greater physiological dysregulation of the haematopoietic and oxygen transport systems, is more accentuated in men.^10^ This dysregulation generates a greater loss of muscle mass and strength, with greater atrophy of type II fibres and repercussions for functional disability over time.^37^ Thus, the dysregulation of the musculoskeletal system compromises the integrity and adequate functioning of other systems, especially the cardiovascular, respiratory, circulatory, and nervous systems. Therefore, it is likely that weakness and slowness share similar mechanisms and, together, contribute to the emergence of disability, especially in men.^37^ A similar process is also seen in women. However, given the greater prevalence of comorbidities, the exacerbation or decompensation of clinical conditions results in a greater energy need, triggering the incidence of exhaustion.^38^ This is in line with the present findings, which highlight exhaustion, together with slowness, as exerting an influence on the incidence of functional disability.

According to the model proposed by Fried *et al*.,^1^ exhaustion is the incapacity to maintain the production, distribution, and use of energy necessary for maintaining the homeostasis of physiological systems. A reduction in the availability of energy can diffusely affect multiple physiological systems, leading to a decline in physical functioning, especially with regards to performing activities that require greater physical endurance. However, exhaustion does not completely capture the multidimensionality of the symptom. The different terminologies used to define this symptom reported by older adults (tiredness, exhaustion, weakness, or low energy), the complexity of the physiopathological mechanisms (involving both physical and mental aspects), the dynamic or isolated nature of fatigue and the lack of a gold standard measure for the evaluation of exhaustion, result in this symptom being overlooked by health professionals and difficult to diagnose.^39^ Therefore, regardless of the terminology used, the low production of energy increases muscle fatigue and, consequently, protein catabolism, which leads to a reduction in muscle mass and strength, affecting the capacity to perform activities of daily living,^40^ which men describe as weakness and women describe as exhaustion.

However, as the construction of exhaustion is based on the CES‐D,^18^ there may be a relation between exhaustion and depression, as both are clinical conditions with a similar nature and converge into a disabling process,^39^ especially for women, among whom the prevalence of depression is higher. Shimada *et al*.^35^ state that frail older people with slow gait and depressive symptoms have a 46% greater risk for the incidence of disability.

Although the Fried *et al*.^1^ model is a construct that is rapidly applied and accepted by the scientific community, the frailty entity may be formed by different combinations of components, which, in the clinical scenario, hinders a more specific intervention for reversing or attenuating the progression of frailty and the disability it causes. Our findings suggest that the components of frailty identified in this study, such as slowness in both sexes, weakness in men and exhaustion in women, can be considered good discriminators for the incidence of disability.

The present study has several strengths. First, standardized tools were used to identify frailty syndrome. Second, the study was conducted with a large representative sample with a long follow‐up period. Third, the generalized linear mixed models were capable of accompanying the dynamic nature of functional disability with the development of frailty over time, encompassing the dynamics of the variables associated with disability in BADL and IADL as well as frailty. Fourth, the comparisons of the individuals included and excluded at baseline identified few differences between these two groups, which demonstrate a low risk of bias in the study. Finally, this is the first study to perform a longitudinal analysis of the incidence trajectories of BADL and IADL disability as a function of changes in frailty status and its components by sex, in individuals without disability and frailty at baseline, in addition to identify which component(s) would be the best discriminator(s) of worse incidence trajectories of disability stratified by sex and adjusted for a wide range of covariates.

However, this study also has limitations that should be acknowledged. First, BADL and IADL were self‐reported. Although this may be a source of bias, methodological studies have shown that self‐reported data have satisfactory validity and are consistent with the results of physical tests. Second, the exclusion of individuals at baseline may have caused some degree of bias. However, we only found significant difference between the included and excluded individuals with regards to alcohol intake not declared, smoking, and the prevalence of hypertriglyceridemia. Finally, the losses to follow‐up may be a source of bias, but this type of bias is inevitable in longitudinal studies involving community‐dwelling older adults.

## Conclusion

Slowness is the main warning sign of functional decline in older adults. As its evaluation is easy, fast, and accessible, screening for this frailty component should be prioritized in different clinical contexts so that rehabilitation strategies can be developed to avoid the onset of disability.

## Conflict of interest

D.C.O., R.O.M., P.C.R.M., A.L.S., M.M.L., M.L.B.D., M.H.N.C., A.S., C.O., and T.S.A. have no conflicts of interest.

## Funding

The present study received funding from the Brazilian fostering agency *Coordenação de Aperfeiçoamento de Pessoal de Nível Superior* [Coordination for the Advancement of Higher Education Personnel (CAPES); Financing code: 001]. The *Conselho Nacional de Desenvolvimento Científico e Tecnológico* [CNPq (National Council of Scientific and Technological Development); concession numbers: 303981/2017‐2 and 303577/2020‐7] and *Fundação de Amparo à Pesquisa do Estado de São Paulo* [FAPESP (State of São Paulo Research Assistance Foundation); process number: 18/13917‐3] support Tiago da Silva Alexandre. ELSA is funded by the National Institute on Aging USA (Grant R01AG017644) as well as by a consortium of governmental departments of the United Kingdom coordinated by the Economic and Social Research Council (ESRC).

## Supporting information


**Table S1.** Socioeconomic, behavioral and biochemical characteristics of included and excluded individuals free of disability and frailty at baseline of ELSA (2004–05).
**Table S2.** Clinical characteristics of included and excluded individuals free of disability and frailty at baseline of ELSA (2004–05).
