# Supplementary material for: Is slowness a better discriminator of disability than frailty in older adults?
Source: J Cachexia Sarcopenia Muscle. 2021 Sep 29;12(6):2069–78. doi: 10.1002/jcsm.12810 (PMC8718056; doi:10.1002/jcsm.12810)
Supplement: Supplementary file 1 — Table S1. Socioeconomic, behavioral and biochemical characteristics of included and excluded individuals free of disability and frailty at baseline of ELSA (2004–05). Table S2. Clinical characteristics of included and excluded individuals free of disability and frailty at baseline of ELSA (2004–05). [file JCSM-12-2069-s001.docx]

**Supplementary Table 1** Socioeconomic, behavioral and biochemical characteristics of included and excluded individuals free of disability and frailty at baseline of ELSA (2004-05)

|  | **BADL** | | | **IADL** | | |
| --- | --- | --- | --- | --- | --- | --- |
|  | **Included**  (n=1.522)  71.2% | **Excluded**  (n=616)  28.8% | **Total**  n=2.138 | **Included**  (n=1.548)  71.1% | **Excluded**  (n=632)  28.9% | **Total**  n=2.180 |
| **Socioeconomic variables** |  |  |  |  |  |  |
| Age, years (SD) | 68.1 ± 6.2 | 68.6 ± 6.3 | 68.2 ± 6.2 | 68.1 ± 6.1 | 68.7 ± 6.3 | 68.3 ± 6.2 |
| Without married life (yes), % | 27.1 | 29.9 | 27.9 | 26.7 | 29.9 | 27.6 |
| Non-white skin color (yes), % | 1.1 | 1.8 | 1.3 | 1.0 | 1.6 | 1.1 |
| Family wealth (quintiles), % | |  |  |  |  |  |
| Highest quintile | 29.7 | 27.1 | 29.0 | 30.0 | 26.7 | 29.1 |
| 2^nd^ quintile | 25.5 | 24.6 | 25.1 | 25.1 | 23.9 | 24.8 |
| 3^rd^ quintile | 20.9 | 19.8 | 20.7 | 21.4 | 21.0 | 21.3 |
| 4^th^ quintile | 14.6 | 15.8 | 14.9 | 14.4 | 16.2 | 14.9 |
| Lowest quintile | 8.2 | 10.8 | 8.9 | 7.9 | 10.5 | 8.6 |
| Not declared | 1.1 | 1.9 | 1.4 | 1.2 | 1.7 | 1.3 |
| Schooling, % | |  |  |  |  |  |
| > 13 years | 30.6 | 26.5 | 29.4 | 30.8 | 26.2 | 29.4 |
| 12–13 years | 24.2 | 24.3 | 24.3 | 24.2 | 23.6 | 24.1 |
| 0–11 years | 45.2 | 49.2 | 46.3 | 45.0 | 50.2 | 46.5 |
| **Behavioral variables** | |  |  |  |  |  |
| Alcohol intake, % |  |  |  |  |  |  |
| ≤ 1 day per week | 13.3 | 13.5 | 13.3 | 13.5 | 13.4 | 13.5 |
| 2-6 days per week | 43.9 | 41.7 | 43.3 | 44.1 | 41.8 | 43.4 |
| Daily | 37.6 | 35.5 | 37.0 | 37.6 | 35.9 | 37.1 |
| Not declared | 5.2* | 9.3* | 6.4 | 4.8* | 8.9* | 6.0 |
| Smoking, % |  |  |  |  |  |  |
| Non-smoker | 42.1 | 35.9 | 40.3 | 41.9* | 35.3* | 40.0 |
| Ex- smoker | 50.3 | 51.1 | 50.5 | 50.5 | 52.5 | 51.1 |
| Smoke | 7.6* | 13.0* | 9.2 | 7.6* | 12.2* | 8.9 |
| Active lifestyle, % |  |  |  |  |  |  |
| Low | 100.0 | 100.0 | 100.0 | 100.0 | 100.0 | 100.0 |
| **Biochemical characteristics** | |  |  |  |  |  |
| Triglycerides (≥150 mg/dL), % | 37.7* | 50.4* | 39.4 | 38.2* | 49.4* | 39.7 |
| Total cholesterol (≥200 mg/dL), % | 74.8 | 79.1 | 75.4 | 74.3 | 77.8 | 74.8 |
| HDL *(*<40 mg/dL M;<50 mg/dL W*),* % | 11.6 | 13.0 | 11.8 | 12.2 | 14.8 | 12.6 |
| LDL (≥100 mg/dL), % | 86.1 | 85.4 | 86.0 | 85.8 | 85.2 | 85.7 |
| Fibrinogen (>3.7 g/l), % | 20.6 | 21.5 | 20.7 | 20.9 | 22.2 | 21.0 |
| Anaemia (<13 g/dL M; <12g/dl W), % | 3.0 | 2.3 | 2.9 | 3.1 | 2.6 | 3.0 |

Note: Data expressed as mean, standard deviation and proportion. ^§^ All individuals with a sedentary lifestyle were excluded at baseline and there were no individuals in the group of vigorous/moderate physical activity. Abbreviations: ELSA – English Longitudinal Study of Ageing; BADL – basic activities of daily living; IADL – instrumental activities of daily living; HDL – high-density lipoprotein; LDL – low-density lipoprotein; M – men; W – women. * Significant difference between sexes (p < 0,05, chi-square test).

**Supplementary Table 2** Clinical characteristics of included and excluded individuals free of disability and frailty at baseline of ELSA (2004-05)

|  | **BADL** | | | **IADL** | | |
| --- | --- | --- | --- | --- | --- | --- |
|  | **Included**  (n=1.522)  71.2% | **Excluded**  (n=616)  28.8% | **Total**  n=2.138 | **Included**  (n=1.548)  71.1% | **Excluded**  (n=632)  28.9% | **Total**  n=2.180 |
| **Clinical conditions** |  |  |  |  |  |  |
| Stroke (yes), % | 2.2 | 3.7 | 2.7 | 2.1 | 3.6 | 2.5 |
| Heart disease (yes), % | 19.2 | 20.6 | 19.6 | 19.4 | 19.9 | 19.6 |
| Cancer (yes), % | 7.6 | 9.2 | 8.1 | 7.4 | 9.0 | 7.8 |
| Lung disease (yes), % | 13.8 | 14.6 | 14.0 | 13.8 | 13.8 | 13.8 |
| Joint disease (yes), % | 28.1 | 27.6 | 27.9 | 28.8 | 28.5 | 28.7 |
| Osteoporosis (yes), % | 8.7 | 10.4 | 9.2 | 7.9 | 9.1 | 8.3 |
| Falls (yes), % | 23.4 | 24.0 | 23.6 | 23.4 | 24.5 | 23.8 |
| Dementia (yes), % | 0.3 | 0.2 | 0.2 | 0.1 | ─ | 0.1 |
| Hypertension (yes), % | 72.9 | 77.6 | 73.9 | 73.3 | 78.6 | 74.4 |
| Diabetes (yes), % | 7.4 | 7.1 | 7.4 | 7.8 | 6.7 | 7.7 |
| Perception of hearing, % | |  |  |  |  |  |
| Good | 82.2 | 84.2 | 82.8 | 82.4 | 84.5 | 83.0 |
| Fair | 14.9 | 12.5 | 14.2 | 14.8 | 12.2 | 14.1 |
| Poor | 2.9 | 3.3 | 3.0 | 2.8 | 3.3 | 2.9 |
| Perception of vision, % |  |  |  |  |  |  |
| Good | 93.8 | 91.7 | 93.2 | 93.5 | 92.4 | 93.2 |
| Fair | 5.1 | 7.0 | 5.6 | 5.5 | 6.7 | 5.9 |
| Poor | 1.1 | 1.3 | 1.2 | 1.0 | 0.9 | 0.9 |
| Depressive symptoms, % |  |  |  |  |  |  |
| No | 98.0 | 97.4 | 97.8 | 98.1 | 97.6 | 97.9 |
| Yes | 1.7 | 1.6 | 1.7 | 1.6 | 1.8 | 1.7 |
| Not declared | 0.3 | 1.0 | 0.5 | 0.3 | 0.6 | 0.4 |
| Mean recall score, points (SD) | 10.3 ± 3.1 | 10.2 ± 3.3 | 10.3 ± 3.2 | 10.3 ± 3.0 | 10.3 ± 3.3 | 10.3 ± 3.1 |
| BMI (kg/m²), % |  |  |  |  |  |  |
| Normal weight (≥ 18.5 and < 25) | 30.2 | 28.9 | 29.8 | 28.8 | 28.0 | 28.6 |
| Overweight (≥ 25 and < 30) | 47.7 | 47.1 | 47.5 | 47.6 | 46.5 | 47.3 |
| Obesity (≥ 30) | 22.1 | 24.0 | 22.7 | 23.6 | 25.5 | 24.1 |

Notes: Data expressed as mean, standard deviation and proportion. (–) missing data. Abbreviations: ELSA – English Longitudinal Study of Ageing; BADL – basic activities of daily living; IADL – instrumental activities of daily living; CES-D *–* Center for Epidemiological Studies-Depression Scale; BMI – body mass index. *Significant difference between sexes (p < 0,05, chi-square test).
